# Anti-CD47 Treatment Stimulates Phagocytosis of Glioblastoma by M1 and M2 Polarized Macrophages and Promotes M1 Polarized Macrophages *In Vivo*

**DOI:** 10.1371/journal.pone.0153550

**Published:** 2016-04-19

**Authors:** Michael Zhang, Gregor Hutter, Suzana A. Kahn, Tej D. Azad, Sharareh Gholamin, Chelsea Y. Xu, Jie Liu, Achal S. Achrol, Chase Richard, Pia Sommerkamp, Matthew Kenneth Schoen, Melissa N. McCracken, Ravi Majeti, Irving Weissman, Siddhartha S. Mitra, Samuel H. Cheshier

**Affiliations:** 1 Division of Pediatric Neurosurgery, Department of Neurosurgery, Lucile Packard Children’s Hospital, Stanford University School of Medicine, Stanford, California, United States of America; 2 Institute for Stem Cell Biology and Regenerative Medicine, Stanford University School of Medicine, Stanford, California, United States of America; 3 Ludwig Center for Cancer Stem Cell Research and Medicine at Stanford, Stanford University School of Medicine, Stanford, California, United States of America; Center for Cancer Research, National Cancer Institute, UNITED STATES

## Abstract

Tumor-associated macrophages (TAMs) represent an important cellular subset within the glioblastoma (WHO grade IV) microenvironment and are a potential therapeutic target. TAMs display a continuum of different polarization states between antitumorigenic M1 and protumorigenic M2 phenotypes, with a lower M1/M2 ratio correlating with worse prognosis. Here, we investigated the effect of macrophage polarization on anti-CD47 antibody-mediated phagocytosis of human glioblastoma cells *in vitro*, as well as the effect of anti-CD47 on the distribution of M1 versus M2 macrophages within human glioblastoma cells grown in mouse xenografts. Bone marrow-derived mouse macrophages and peripheral blood-derived human macrophages were polarized *in vitro* toward M1 or M2 phenotypes and verified by flow cytometry. Primary human glioblastoma cell lines were offered as targets to mouse and human M1 or M2 polarized macrophages *in vitro*. The addition of an anti-CD47 monoclonal antibody led to enhanced tumor-cell phagocytosis by mouse and human M1 and M2 macrophages. In both cases, the anti-CD47-induced phagocytosis by M1 was more prominent than that for M2. Dissected tumors from human glioblastoma xenografted within NOD.Cg-*Prkdc*^*scid*^ *Il2rg*^*tm1Wjl*^/SzJ mice and treated with anti-CD47 showed a significant increase of M1 macrophages within the tumor. These data show that anti-CD47 treatment leads to enhanced tumor cell phagocytosis by both M1 and M2 macrophage subtypes with a higher phagocytosis rate by M1 macrophages. Furthermore, these data demonstrate that anti-CD47 treatment alone can shift the phenotype of macrophages toward the M1 subtype *in vivo*.

## Introduction

Glioblastoma (GBM, WHO grade IV) is the most aggressive and common malignant primary brain tumor in adults. The current standard of care of radiotherapy plus concomitant and adjuvant temozolomide can increase survival, but median survival continues to be 15–16 months [1–3]. Novel therapeutics remain elusive despite an improved understanding of the GBM microenvironment.

Macrophages have demonstrated promising anti-tumor potential given their inherent ability to phagocytose damaged cells [4]. The human body’s natural immune response is thought to have the means to selectively and effectively target GBM, and the development of immunotherapies against GBM using dendritic cells, T cells, or natural killer cells are currently underway [5–7]. However, macrophages and microglia (tumor-associated macrophages/microglia, TAMs), which can comprise up to 30% of the bulk of tumors, are the dominant immune cells within many malignancies [8]. Thus, TAMs are an intriguing immune cell target for treatment in malignant gliomas.

Macrophage response to foreign pathogens and biological insults is known to be heterogeneous, and a continuum of activation patterns between pro-inflammatory/classic (M1) and anti-inflammatory/alternatively activated (M2) macrophages exists. M1 macrophages facilitate the removal of bacteria and damaged cells and have been shown to have anti-tumorigenic properties, while M2 macrophages promote tissue repair and tumor proliferation [9–12]. Both subtypes are known to coexist within the glioma microenvironment. Generally, an increased presence of M1 TAMs correlates with lower grade glioma, while a higher M2 presence associates with increased glioma growth and decreased glioma patient survival [13–17]. These and other data suggest that the M1 macrophages counter tumor growth, whereas M2 macrophages support/promote tumor growth. Thus, targeted immune-modulation to incorporate or amplify M1 macrophage activity and/or reduce M2 activity in GBM may improve outcomes.

Recently, CD47 has been identified as a “don’t eat me” signal on tumor cells [4, 18, 19]. CD47 expression on tumor cell membranes and subsequent binding to signal-regulatory protein alpha (SIRPα) on macrophages lead to a signal cascade within the macrophages that inhibits macrophage-mediated tumor cell phagocytosis [20, 21]. Recent findings by Bhoukari et al. implicate additionally a tumor-suppressor protein deregulation in astrocytoma cell lines when CD47 is overexpressed [22] which sheds light on a possible tumor-propagating epigenetic mechanism of CD47 overexpression. Disruption of the CD47-SIRPα axis by monoclonal antibodies results in enhanced phagocytosis of both solid and hematopoietic tumor cells, including increased phagocytosis of GBM cells *in vitro* and significant anti-tumor activity *in vivo* [19, 23]. Furthermore Willingham et al. have shown that anti-CD47 blockade is capable of re-educating TAMs from a tumor-promoting role to an anti-tumor one by inducing TAMs, isolated from breast, bladder, and liver cancer xenografts, to phagocytose tumor cells [19]. The nature of the macrophages with respect to M1 versus M2 in these xenografted tumors were not determined in the previous study. Furthermore, to what extent M1 versus M2 macrophage polarization affects phagocytosis of tumor cells in the setting of anti-CD47 treatment has yet to be evaluated. Here we quantify the rate of phagocytosis for M1 and M2 macrophages *in vitro* and observe a larger increase in the phagocytosis rate by M1 macrophages, relative to that by M2 macrophages, however, M2 macrophage phagocytosis of tumor cells was significantly increased by anti-CD47 treatment versus control. We also show that upon tumor cell opsonisation and/or the disruption of CD47-SIRPa interactions by blocking anti-CD47 treatment, the *in vivo* tumor microenvironment reflects a potentially beneficial M1-dominant profile, strongly suggesting either the re-education of M2 TAMs into M1 macrophages or the enhanced recruitment of M1 macrophages from the periphery is occurring in this setting.

## Materials and Methods

### Ethics statement

Human adult and pediatric brain tumor tissue samples were obtained at Stanford University Medical Center and Lucile Packard Children’s Hospital (Stanford, CA) in accordance with institutional review board protocols (http://humansubjects.stanford.edu) and the administrative panel on human subjects research **(IRB protocol ID 18672; IRB Number 350: Panel 3)**. All patients or their next of kin gave a written informed consent for tumor biopsy collection and signed a declaration permitting the use of their biopsy specimens in scientific research. IRB deemed protocol as “exempt” since tissue was acquired through the Stanford tissue bank (http://tissuebank.stanford.edu) and all patient identifying information was removed and tissue was coded for identification. All protocols for the experiments involving mice, the handling of the animals and the surgical procedures were done in accordance with the Institutional Animal Care and Use Committee (IACUC) approved the protocol number 26548 and Assurance Number A3213-01. Mice were housed in a vivarium accredited by the American Association for Accreditation of Laboratory Animal Care.

### Mouse management

NOD.Cg-*Prkdc*^*scid*^ *Il2rg*^*tm1Wjl*^/SzJ (NSG) mice were housed in specific pathogen-free conditions at a barrier facility at the Lokey Stem Cell Building (SIM1) at Stanford School of Medicine (Stanford, CA). All animal handling, surveillance, and experimentation were performed in accordance with and approval from the Stanford University Administrative Panel on Laboratory Animal Care (Protocol 26548).

### Primary cell lines and cell cultures

The malignant glioma cell line D270 was generously provided by Gerry Grant (Duke University, Durham, NC) [24]. Pathologies of patient tumors were confirmed upon histologic analysis by board-certified neuropathologists prior to further analysis. Briefly, tumors were enzymatically dissociated at 37°C for 30 minutes with collagenase-IV (CLS-4: Worthington) and DNAse1 in a solution containing Hank’s Balanced Salt Solution (HBSS) with Ca^2+/^Mg^2+^, non-essential amino acids, sodium pyruvate, HEPES, glutamine and antibiotic-antimycotic (Corning Inc., Corning, NY, USA). Following mechanical trituration, cells were filtered and subjected to a 0.9 M sucrose gradient centrifugation to dispose of dead cells and myelin. Upon ACK-lysis for removal of erythrocytes (Gibco Life Technologies, Carlsbad, CA, USA), cells were washed, counted and plated in a density of 1 x 10^5^/ml in glioblastoma stem cell medium, consisting of 50% Neurobasal-A (Gibco Life Technologies), 50% DMEM/F12, non-essential amino acids, sodium pyruvate, sodium bicarbonate, HEPES, glutamine, antibiotic-antimycotic (Corning Inc.), 20 ng/mL human recombinant EGF, 20 ng/mL human recombinant FGF (Shenandoah Biotech, Warwick, PA, USA), heparin sulfate (Sigma-Aldrich, St. Louis, MO, USA) and B27-supplement without Vitamin A (Gibco Life Technologies). Cells were allowed to form spheres and were grown for 2 passages. A list of the generated cell lines as well as the clinical characteristics of the tumors is summarized in Table 1.

**Table 1:** Cell lines used for *in vitro* and *in vivo* experiments, tissue of origin.

Name	Designation	Researcher	Histology	*In Vivo*	*In Vitro*
GBM1	SU-GBM005	Cheshier Laboratory, Stanford University	GBM WHO Grade IV		X
GBM2	SU-GBM066	Cheshier Laboratory, Stanford University	GBM WHO Grade IV		X
GBM3	SU-GBM045	Cheshier Laboratory, Stanford University	GBM WHO Grade IV		X
GBM4	D270	Grant Laboratory, Duke University	GBM WHO Grade IV	X	X
GBM5	SU-GBM044	Cheshier Laboratory, Stanford University	GBM WHO Grade IV	X	
GBM6	SU-GBM 062012	Cheshier Laboratory, Stanford University	GBM WHO Grade IV		X
PGBM1	SU-PHGG002	Cheshier Laboratory, Stanford University	Pediatric GBM WHO Grade IV		X
NPC 3643	Received March 2012	StemExpress, Pleasantville, CA	Fetal neural progenitor cells (NPC)		X

Human fetal brain tissue from gestational week 16 was obtained from a commercial source (StemExpress, Placerville, CA, USA) and dissociated to single cells using TryPLE (Life Technologies) and cultured in neurosphere growth media additionally supplemented with human recombinant LIF (Shenandoah Biotech) [25].

### Human PBMC derived macrophage preparation

Human peripheral blood mononuclear cells (PBMCs) were collected from venous blood of healthy volunteers, and in leukocyte reduction chambers, diluted 2x with HBSS (Corning Inc.) and separated via Ficoll density gradient (GE Healthcare, Sunnyvale, CA, USA). CD14+ monocytes were positively selected to >95% purity by MACS using anti-CD14 microbeads (Miltenyi, Biotec, Auburn, CA, USA), then plated at 1 x 10^7^/ml in 150 x 25 mm tissue culture plates in RPMI 1640 with 10% FBS, antibiotic-antimycotic, glutamine, and HEPES (Corning Inc.).

### Mouse bone marrow-derived macrophage preparation

Mouse macrophages were obtained from 7–11-week-old NSG (NOD.Cg-*Prkdc*^*scid*^ *Il2rg*^*tm1Wjl*^/SzJ) mouse bone marrow. Briefly, mice were euthanized in a CO_2_ chamber and *femura* and *tibiae* were isolated. The bones were kept in ice-cold PBS and sterilized in 70% ethanol. By flushing them with mouse macrophage medium (IMDM with 10% FBS, 1x penicillin/streptomycin, 200 mM glutamine, and 25 mM HEPES, all from Corning Inc.), bone marrow cells were gathered and plated at 1 x 10^6^/ml in 100 x 25 mm petri dishes in mouse macrophage medium.

### Polarization protocol

To generate M0 or M2 macrophages, sorted monocytes or bone-marrow cells were treated for 7 days with either recombinant human or mouse macrophage colony-stimulating factor (M-CSF; 25 ng/mL). M2 polarization was achieved by further treatment on Days 5 and 6 with IL-4 (20 ng/mL) and IL-13 (20 ng/mL). To generate M1 macrophages, sorted monocytes or bone marrow cells were treated for 7 days with either recombinant human or mouse granulocyte macrophage colony-stimulating factor (GM-CSF; 5 ng/mL). M1 polarization was achieved with further treatment on Day 5 by interferon gamma (IFN-γ; 20 ng/mL) stimulation for 1 hour, followed by lipopolysaccharide (LPS) for 48 hours (100 ng/mL; Sigma-Aldrich). Unless otherwise stated, all cytokines were purchase from Shenandoah Biotech.

### Quantitative real-time PCR

Total RNA was isolated from murine macrophages using the TRIZol reagent (Life Technologies) according to the manufacturer’s instructions. cDNA was synthesized from 750 ng total RNA and reverse transcribed with the RT^2^ First Strand Kit (Qiagen, Valencia, CA, USA) according to the manufacturer’s instructions. Real-time PCR was performed using RT^2^ SYBR^®^ Green ROX ^™^ qPCR Mastermix (Qiagen). Primer sets for *Nos1*: (F) 5’-GTT GCT GAA CTT CCA GTC ATT G-3’, (R) 5’-AGC CCT CAC CTA CTT CCT G-3’ and *Mrc1*: (F) 5’-AGT GTT GAT GTC AGT GTG AGC-3’, (R) 5’-GAA TGG AAG AGT CAG TGT GGT-3’ were purchased from Integrated DNA Technologies (IDT, Coralville, IA, USA). *Hprt*: (F) 5’-CAC CCT TTC CAA ATC CTC AG-3’ (R) 5’-CTC CGT TAT GGC GAC CC-3’ was used as a housekeeping gene. The 2^ΔΔ*C*t^ method was used to calculate fold changes in gene expression normalized to *Hprt*.

### Analysis of cytokines and chemokines in macrophage culture supernatants

This assay was performed in the Human Immune Monitoring Center at Stanford University. Human 51-plex and Mouse 26-plex kits were purchased from Affymetrix (Santa Clara, CA, USA) and used according to the manufacturer’s recommendations with modifications as described below. Briefly, samples were mixed with antibody-linked polystyrene beads on 96-well filter-bottom plates and incubated at room temperature for 2 hours followed by overnight incubation at 4°C. Room temperature incubation steps were performed on an orbital shaker at 500–600 rpm. Plates were vacuum-filtered and washed twice with wash buffer, and then incubated with biotinylated detection antibody for 2 hours at room temperature. Samples were then filtered and washed twice as above and resuspended in streptavidin-PE. After incubation for 40 minutes at room temperature, two additional vacuum washes were performed, and the samples resuspended in reading buffer. Each sample was measured in duplicate. Plates were read using a Luminex 200 instrument with a lower bound of 100 beads per sample per cytokine.

### Flow cytometry analysis

The following antibodies were used for analyses of mouse bone marrow-derived mouse macrophages and human xenografts in mice: CD11b PE-Cy7, F4/80 Alexa Fluor 700, CD80 Alexa Fluor 647, CD206 PE (BioLegend, San Diego, CA, USA). Antibodies for analysis of PBMC-derived human macrophages included CD11b Alexa Fluor 647, CD14 APC-Cy7, CD80 PE-Cy7, CD163 PE (BioLegend). DAPI stain (Invitrogen, Eugene OR, USA) was added to exclude dead cells.

Acquisition was performed on an LSRII Fortessa flow cytometer (BD-Biosciences, Franklin Lakes, NJ, USA) and sorting was performed on a FACSAria II (BD-Biosciences). Data analysis was performed using FlowJo Version 9.6.4 (Tree Star, Ashland, OR, USA). Live singlets were gated using FSC-W/FSC-H. Gates were drawn by using Fluorescent Minus One (FMO) control tubes. Macrophages were separated from tumor cells by their expression of CD11b and CD14 for human as well as CD11b and F4/80 for mouse macrophages.

### Therapeutic antibodies

Hu5F9-G4 [26] was constructed using CDR grafting from a mouse-anti-human CD47 antibody, clone 5F9. Since Hu5F9-G4 activity is primarily dependent on blocking the CD47-SIRP-alpha interaction, a human IgG4 scaffold was selected to minimize the recruitment of Fc-dependent effector functions such as antibody dependent cellular cytotoxicity (ADCC), antibody-dependent cellular phagocytosis (ADCP), and complement-dependent cytotoxicity (CDC). The mechanism of action does not require these functions, and their presence may increase toxicity against normal cells. Hu5F9-G4 is engineered using a human kappa and IgG4 isotype with the Ser228Pro substitution to reduce Fab arm exchange. Cetuximab (Bristol-Meyers Squibb) was purchased from the Stanford clinic.

### *In vitro* phagocytosis assay

Dissociated tumor cultures were CFSE or calcein-labeled (BioLegend) and incubated with either mouse or human macrophages in serum-free IDMEM, with or without 10 μg/mL anti-CD47 monoclonal antibody (Hu5F9, IgG4) at 37°C for 2 hours (coincubation). The tumor cell to macrophage ratio was 2:1. In some instances, tumor cells or macrophages alone were pretreated with anti-CD47 antibody for 30 min and the antibody was washed out twice.

*Preparation of tumor cells*: For carboxyfluorescein diacetate succinimidyl ester (CFSE) staining, an 8.97 μM solution of CFSE in HBSS was prepared. Cells were resuspended in 1 mL of staining solution per 1 x 10^6^ cells and incubated for 10 minutes in a 37°C water bath. Subsequently, excessive CFSE was quenched using ice-cold HBSS. Stained cells were resuspended in serum-free Icove’s DMEM at a cell concentration of 4 x 10^6^ cells/ml. Alternatively, tumor cells were stained with calcein. A 5.27 nM solution of calcein in HBSS was prepared. Cells were resuspended in 1 mL of staining solution per 1 x 10^6^ cells and incubated for 10 minutes in a 37°C water bath. Subsequently, excessive calcein was quenched using ice-cold HBSS. 25 μL of tumor cell suspension (100,000 cells) per well were dispensed in an ultra low-attachment 96 well plate.

#### Preparation of macrophages

Human PBMC-derived macrophages and murine BM-derived differentially polarized macrophages were washed twice with HBSS or PBS, respectively. Cells were detached from the tissue culture plates using TrypLE and cell lifters. Cells were washed with ice-cold HBSS thrice and the cell number was adjusted to 2 x 10^6^ cells/mL in serum-free Icove’s DMEM. 25 μL of the macrophage cell suspension (50,000 cells) were added to the tumor cells in the respective wells of the 96-well plate and incubated for 2 hours at 37°C. Technical triplicates of all conditions were prepared to compare the macrophage subtype activity from a single host towards a given tumor line. For experiments to specifically isolate the contribution of FcgR-mediated phagocytosis, tumor cells and macrophages were pre-incubated with Fc Block (BD Biosciences) containing recombinant Fc protein to block Fc receptors on macrophages to eliminate Fc mediated ADCP prior to mixing the cells for phagocytosis. Tumor cells and macrophages were pre-incubated with 10ug/ml of recombinant Fc protein for 60 minutes on ice. Cells were spun down only supernatant removed and pelleted cells prepared for phagocytosis assay as described above.

Staining of macrophages was done directly after the phagocytosis assays in the 96-well plates utilized for the assay. The respective antibody master mix was added directly to the wells after the phagocytosis assay was finished. The master mix additionally contained mouse IgG (1 μg per 1 x 106 cells) to block unspecific binding of antibodies to Fc-receptors of macrophages. Cells were incubated with the antibodies for 30 minutes on ice in the dark. Subsequently, cells were washed twice with FACS buffer and DAPI staining was performed to enable exclusion of dead cells from analysis. Cells were analyzed with a BD LSR Fortessa using a high throughput sampler. Gates were placed according to unstained and fluorescence-minus-one (FMO) controls. Fluorescently labeled antibodies targeting macrophage markers CD11b and CD14 (human) or CD11b and F4/80 (mouse) were used to identify the macrophage population (S1 and S2 Figs) Macrophages that had successfully phagocytized tumor cells were also positive for the CFSE or calcein stain. Phagocytosis for each macrophage subtype was quantified by the percentage of CFSE+ events among CD80+ (mouse and human M1 macrophages), CD206+ (mouse M2 macrophage) or CD163+ (human M2 macrophage events.

### Orthotopic xenografts for *in vivo* analysis

Early passage spheres (5^th^ passage) of GBM4 and GBM5 were transduced with GFP and luciferase encoding lentivirus (pEF1-GFP-E2A-FLuc, System Biosciences, Mountain View, CA, USA), expanded in glioblastoma stem cell media and double sorted to obtain a 100% luciferase-expressing population. The selected population was expanded in glioma stem cells media and orthotopically injected to the site of tumor resection. In brief, 6–10-week-old mice were anesthetized with 3% isoflurane (Minrad International, Buffalo, NY, USA) in an induction chamber. Anesthesia on the stereotactic frame (David Kopf Instruments, Tujunga, CA, USA) was maintained at 2% isoflurane delivered through a nose adaptor. A burr hole was placed 2 mm lateral and 3 mm posterior of bregma. A blunt-ended needle (75N, 26s/2”/2, 5 μL; Hamilton Co., Reno, NV, USA) was lowered into the burr hole to a depth of 4 mm below the dura surface and retracted 1 mm to form a small reservoir. Using a microinjection pump (UMP-3; World Precision Instruments, Sarasota, FL, USA), 10^5^ cells were injected in a volume of 4 μL at 35 nL/second. After leaving the needle in place for 2 minutes, it was retracted at 1 mm/min.

Tumor formation was followed by bioluminescence imaging on IVIS spectrum (Caliper Life Science, Hopkinton, MA, USA) and quantified with Live Image 4.0 software (Living Image, PerkinElmer, Waltham, MA, USA). Engrafted mice, based on a region of interest photon flux rate of >10^5^/s after 14 days (GBM4) and 35 days (GBM5), were then treated with intraperitoneal injections of PBS or 250 μg of the anti-CD47 antibody for 1 week daily (GBM4) or 30 days on alternating days (GBM5), with treatment schedule based on the xenografted mice’s time to morbidity. In the case of GBM4, n = 2 mice per group were analyzed, and n = 6 for GBM5. Mice were sacrificed and the tumor mass was dissected and dissociated for subsequent flow cytometer analysis. For survival and efficacy analysis, 1.5 x 10^5^ GBM5 cells were orthotopically injected and treatment initiated after 35 days. Mice were monitored until endpoint.

### Statistical analysis

Statistical analyses were performed using Graph Pad Prism Software (GraphPad, San Diego, CA, USA). Differences in means as determined by paired Student’s t-test was considered statistically significant at p < 0.05. Kaplan Meier survival curves were assessed by log-rank analysis.

## Results

### Flow-cytometry profile and gene expression of mouse M1 and M2 macrophages

Using a modified protocol based on a published report, we were able to achieve an efficient polarization with >95% subtype differentiation [27, 28]. Bone marrow-derived mouse macrophages, differentiated by either GM-CSF or M-CSF strongly expressed CD11b and F4/80 (S1A Fig). GM-CSF cultured mouse macrophages subsequently stimulated with IFN-γ/LPS were CD80^high^ and CD206^mid^_,_ as described previously [27]. Comparatively, M-CSF differentiated mouse macrophages stimulated with IL-4/IL-13 exhibited a CD80^mid^ and CD206^high^ profile, previously seen with mouse M2 macrophages (S3A Fig) [28]. To further confirm polarization, qPCR analysis of mRNA expression levels was performed. Inducible nitric oxide synthase 1 (*Nos1*) was elevated in IFN-γ/LPS polarized mouse macrophages, whereas mannose receptor 1 (*Mrc1* or CD206) was upregulated in IL-4/IL-13 polarized mouse macrophages (S3B Fig) [29, 30]. Therefore, we term IFN-γ/LPS polarized mouse macrophages as “M1” and IL-4/IL-13 polarized cells as “M2.”

### Flow-cytometry profile and cytokine expression of human M1 and M2 macrophages

Similarly, either GM-CSF- or M-CSF-differentiated peripheral-blood derived human macrophages were found to strongly express CD11b and CD14 (S1B Fig). As expected, human macrophages polarized with IFN-γ/LPS were CD80^high^ and CD163^low^_,_ consistent with M1 macrophages [28]. By contrast, human macrophages polarized with IL-4/IL-13 were found to be CD80^mid^ and CD163^high^, consistent with M2 cells (S4A Fig) [31]. The levels of inflammatory cytokines IL-1ß, TGF-α, IL-6, IL-10, and IL-12P40, were elevated in culture media of IFN-γ/LPS-treated human macrophages relative to those of IL-4/IL-13-treated cells (S4B Fig) [28].

### CD47 expression of tumor cell lines

CD47 expression on the tumor cell lines was measured by FACS analysis and isotype-matched control antibodies. All tumor cell lines as well as the neural stem cell line expressed CD47 at a high level (Table 2, S7 Fig). GBM 4 displayed the highest CD47 expression in comparison to all other cell lines.

**Table 2:** CD47 expression of the tested cell lines.

Tumor cell line	CD47 expression (MFI), isotype corrected
GBM1	8128
GBM2	10000
GBM3	9650
GBM4	35584
GBM5	10432
PHGG	12416
NSC	8384

### Phagocytosis of GBM cells by mouse M1 and M2 macrophages *in vitro*

**Fig 1:**
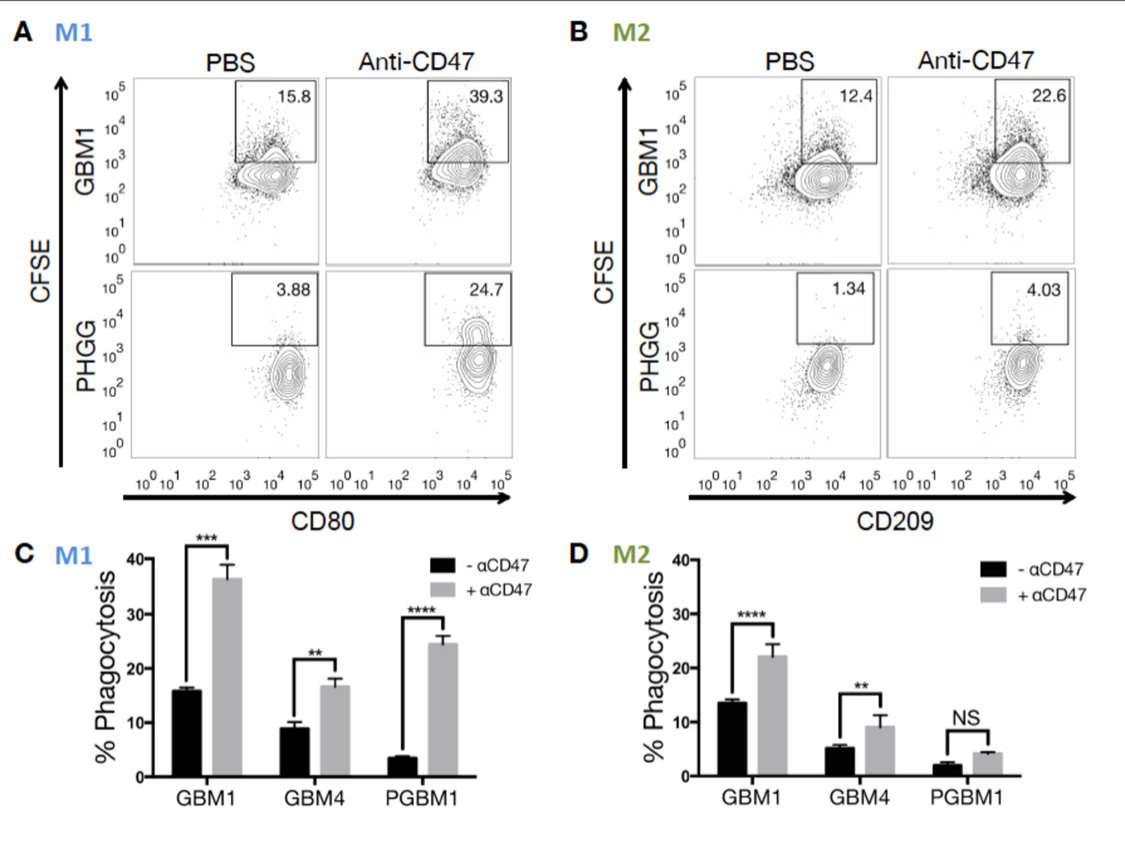
Differential phagocytosis rate of mouse M1 and M2 macrophages toward various human glioma cells upon CD47-SIRPα disruption. (A) Representative flow cytometric phagocytosis assay of mouse M1 macrophages against CSFE-labeled GBM1 and PGBM1 cells. The percentage of CSFE+CD11b+CD80^high^ live singlets was measured and compared between untreated and anti-CD47 antibody-treated co-cultures. (B) Representative flow cytometric phagocytosis assay of mouse M2 macrophages against CSFE-labeled GBM1 and PGBM1 cells. The percentage of CSFE+CD11b+CD206^high^ live singlets was measured and compared between untreated (left column) and anti-CD47 antibody-treated (right column) co-cultures. (C) Bar graph demonstrating the change in phagocytosis rates by mouse M1 macrophages towards individual co-incubated cell lines (GBM1, GBM4 and PGBM1) -/+ anti-CD47 (significant difference in means of technical triplicates indicated by * p ≤ 0.05, ** p ≤ 0.01, *** p ≤ 0.001, **** p ≤ 0.0001, multiple t-tests). (D) Bar graph demonstrating the change in phagocytosis rates by mouse M2 macrophages towards individual co-incubated cell lines (GBM1, GBM4 and PGBM1) -/+ anti-CD47 (significant difference in means of technical triplicates indicated by * p ≤ 0.05, ** p ≤ 0.01, *** p ≤ 0.001, **** p ≤ 0.0001, multiple t-tests).

We then analyzed whether mouse M1 or M2 macrophages had different efficiency of phagocytosis towards GBM cells (S1C Fig). Tumor cells were labeled with CFSE (S1 Fig) or, in a validation experiment, with calcein and co-incubated with macrophages in a 2:1 ratio for two hours. (S2A–S2D Fig) Three different primary tumor cell lines, GBM1, GBM4 and PGBM1, were offered to either mouse M1 or M2 macrophages as targets (Fig 1A–1D, S5A Fig). Upon treatment of the co-cultured cells with 10 μg/mL anti-CD47 antibody, both M1 and M2 macrophages displayed significantly increased mean phagocytosis rates relative to controls in each individual cell line (Table 3). In summary, phagocytosis for mouse M1 macrophages increased by 16% (p = 0.07) compared to 4% for M2 macrophages (p = 0.09, S5B Fig). Mouse M1 phagocytosis rates were higher than in M2 macrophages for all lines (Table 3).

**Table 3:** M1 and M2 macrophage phagocytosis rates after anti-CD47 treatment. Data are means of 3 replicates per tumor cell line.

	M1	M2	P-value
**Mouse Macrophages**			
**GBM1**	36.16	22.07	0.0024**
**GBM4**	16.57	9.02	0.008**
**PGBM1**	24.43	14.3	< 0.0001****
**Human Macrophages**			
**GBM1**	44.93	33.17	0.0011**
**GBM2**	68.43	62.87	0.0538
**GBM3**	58.13	51.63	0.183
**GBM4**	7.93	5.5	0.0364*
**PGBM1**	82.8	35.73	< 0.0001****

* p ≤ 0.05

** p ≤ 0.01

*** p ≤ 0.001

**** p ≤ 0.0001

### Phagocytosis of GBM cells by human M1 and M2 macrophages *in vitro*

**Fig 2:**
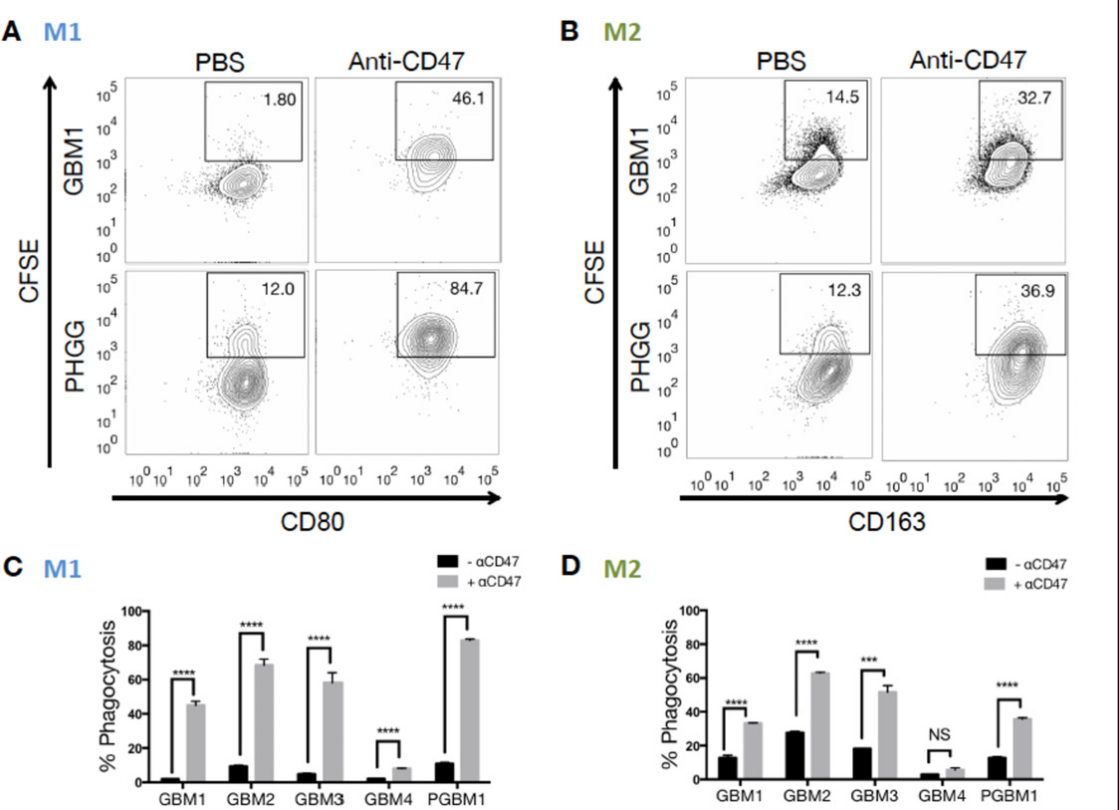
Differential phagocytosis rate of human M1 and M2 macrophages towards various human glioma cells upon CD47-SIRPα disruption. (A) Representative flow cytometric phagocytosis assay of human M1 macrophages against CSFE-labeled GBM1 and PGBM1 cells. The percentage of CSFE+CD11b+CD80^high^ live singlets was measured and compared between untreated (left column) and anti-CD47 antibody-treated (right column) co-cultures. (B) Representative flow cytometric phagocytosis assay of human M2 macrophages against CSFE-labeled GBM1 and PGBM1 cells. The percentage of CSFE+CD11b+CD163^high^ live singlets was measured and compared between untreated and anti-CD47 antibody-treated co-cultures. (C) Bar graph demonstrating the change in phagocytosis rates by human M1 macrophages towards individual co-incubated (GBM1-4 and PGBM1) tumor cells -/+ anti-CD47 (significant difference in means of technical triplicates indicated by * p ≤ 0.05, ** p ≤ 0.01, *** p ≤ 0.001, **** p ≤ 0.0001, multiple t-tests). (D) Bar graph demonstrating the change in phagocytosis rates by human M2 macrophages towards individual tumor cell (GBM1-4 and PGBM1 -/+ anti-CD47 (significant difference in means of technical triplicates indicated by * p ≤ 0.05, ** p ≤ 0.01, *** p ≤ 0.001, **** p ≤ 0.0001, multiple t-tests).

We used the same *in vitro* assay to evaluate whether human M1 and M2 macrophage phagocytosis activities differed toward primary GBM lines. Five different primary tumor cell lines, GBM1-4 and PGBM1, were offered to either human M1 or M2 macrophages as targets (Fig 2, S5B Fig). As before, to evaluate the responsiveness of these M1 and M2 macrophages to anti-CD47 treatment, we administered anti-CD47 antibodies to co-cultures. Both M1 and M2 macrophages showed increased phagocytosis of all tumor lines evaluated (Fig 2). The mean phagocytosis rate of human M1 macrophages toward all tumor cells increased significantly upon anti-CD47 treatment (overall increase 47%, p = 0.015, paired t-test, Fig 2A and 2C, S5B Fig). A significant increase of mean phagocytic activity toward almost all tumor cells could also be observed in M2 macrophages (overall increase 24%, p = 0.015, paired t-test, Fig 2B and 2D, S5B Fig). The resulting human M1 macrophage phagocytosis rates after anti-CD47 treatment were significantly higher than those observed in human M2 macrophages in 3 out of 5 tumors (Table 3). However, in GBM2 and GBM3 where we did not see a statistically significant increase in M1 macrophage phagocytosis, we consistently observed a higher phagocytosis rate in M1 macrophages.

Three primary tumor lines were additionally co-cultured with unpolarized or human M0 macrophages displaying a mean phagocytosis rate of 18.3% at baseline. Anti-CD47 treatment of co-cultured human M0 macrophages with these tumor cells yielded a significantly higher mean phagocytosis rate of 46.7% (difference 28.3%, p = 0.0006, paired t-test, S6A Fig). When neural progenitor cells were offered as targets, phagocytosis by M1 or M2 subsets after anti-CD47 treatment was less than that observed towards glioblastoma (S6B and S6C Fig). This result indicates that the polarization state of macrophages does not affect the specificity of anti-CD47 against tumor cells.

**Fig 3:**
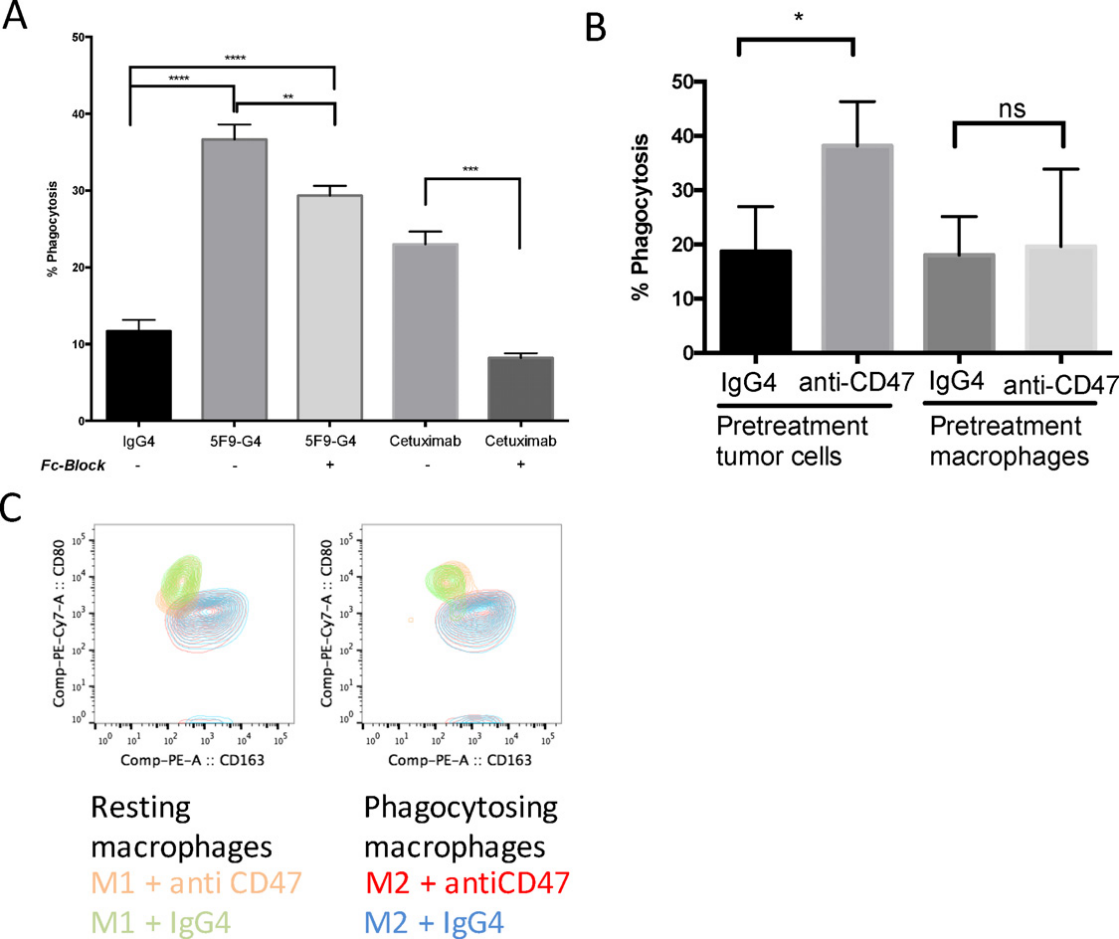
Influence of anti-CD47 pretreatment of tumor cells or pretreatment of macrophages on phagocytosis; polarization properties of M1 and M2 macrophages after anti-CD47 treatment in vitro. (A) Bar graph demonstrating Fc-receptor contribution during anti-CD47 treatment. Anti-CD47 mediated phagocytosis is marginally reduced A monoclonal antibody against EGFR (Cetuximab) was used as an inductor of antibody-dependent cellular phagocytosis (ADCP) which is abrogated upon pre-blocking with an Fc-Blocking peptide. (significant difference in means of technical triplicates indicated by * p ≤ 0.05, ** p ≤ 0.01, *** p ≤ 0.001, **** p ≤ 0.0001, multiple t-tests). (B) Bar graph demonstrating the change in phagocytosis rates by human M0 macrophages towards GBM6 -/+ anti-CD47 pretreatment of tumor cells or pretreatment of macrophages vs. IgG4 isotype-matched control antibody (significant difference in means of technical triplicates indicated by * p ≤ 0.05, multiple t-tests). (C) Overlay contour plots of either resting human M1 or M2 macrophages (CD11b+ CD14+) treated with anti-CD47 antibody or control IgG4 and phagocytosing M1 and M2 macrophages.

To appreciate the effect that Fc-receptor mediated opsonization can have on coinciding Sirp-CD47 disruption on phagocytosis, we performed additional phagocytosis assays with Fc-blocked monoclonal antibodies toward GBM primary lines, thereby reducing nonspecific IgG binding. This strategy of limiting the Fc-receptor contribution continued to yield a phagocytosis rate that was significantly higher than that of IgG4-only control, however it was significantly albeit marginally decreased from that of the unblocked antibody (Fig 3A). To examine how IgG subclass may affect antibody-mediated cellular phagocytosis (ADCP) effects and CD47-Sirp disruption-induced phagocytosis, we treated glioblastoma cells with an IgG1-antibody against an unrelated target, epidermal growth factor receptor (EGFR, cetuximab). Unlike after Fc-receptor blockade of Hu5F9-G4, where a significant contribution of CD47-Sirp disruption towards the overall phagocytosis persisted, the ADCP-effect of Cetuximab significantly decreased upon blocking Fc receptors on macrophages. To detect the baseline antibody-mediated cellular cytotoxicity, we performed an additional comparison of tumor cells (GBM6) and macrophages (M0) pretreated with anti-CD47 or isotype-matched antibody (mouse IgG4) before coincubation. Only pretreating tumor cells with Hu5F9-G4, and not the macrophages, resulted in an increase of phagocytosis (Fig 3B). Pretreatment of macrophages and subsequent wash-out of the antibody did not influence tumor cells phagocytosis (Fig 3B).

We further investigated, whether anti-CD47 treatment is able to induce a polarization shift from M2 to M1 macrophages or from M1 to M2 macrophages *in vitro*. Resting M1 and M2 macrophages did not change their surface receptor profile upon anti-CD47 treatment compared to IgG4 isotype treatment (Fig 3C). Phagocytosing M1 and M2 macrophages equally did not show a shift in their surface receptor profile upon anti-CD47 antibody treatment (Fig 3C).

### *In vivo* treatment with anti-CD47 increases the presence of mouse M1 macrophages in the tumor microenvironment

**Fig 4:**
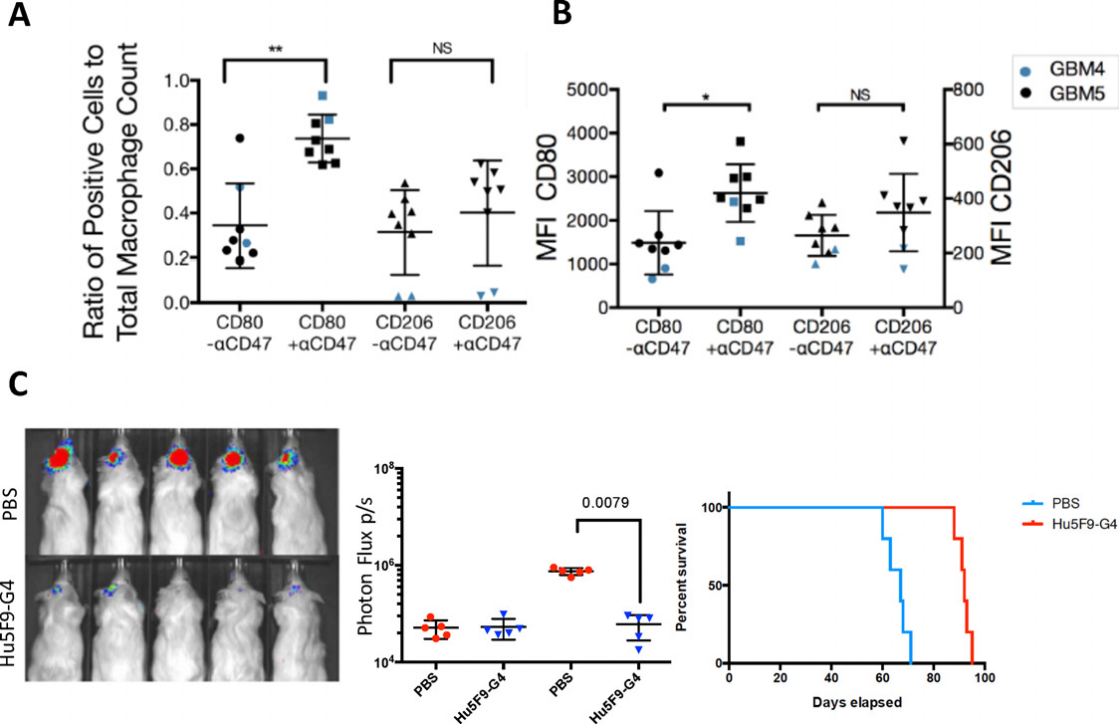
*In vivo* analysis of tumor-associated macrophage polarization upon anti-CD47 treatment; bioluminescence and survival analysis of treated mice. (A) Ratio of CD80 and CD206 positive cell count per total macrophage count in untreated and anti-CD47-treated mice (p = 0.0054 for CD80 and 0.164 for CD260, paired t-test). (B) Median fluorescent intensity (MFI) measurement of CD80-AF647 and CD206-PE (p = 0.0002 for CD80 and 0.423 for CD206, paired t-test). (C) Bioluminescence in vivo imaging data (left panel), photon flux values at days 21 and 50 (middle panel) and Kaplan-Meier analysis of mice grafted with GBM5 and treated with Hu5F9-G4 (right panel, 250 μg/dose, every other day, starting at week 3; n = 5 per group, p = 0.0018, log-rank analysis). Legend: blue shapes: mice engrafted with GBM4, black shapes: mice engrafted with GBM5.

Last, we investigated whether anti-CD47 treatment *in vivo* changed the macrophage polarization profile toward a favorable anti-tumorigenic microenvironment. Mice that had been orthotopically xenografted with primary brain tumor cells from a low-passage primary glioma line (GBM5) or a different, highly aggressive glioma cell line (GBM4), and confirmed by bioluminescence imaging, were subsequently treated with either anti-CD47 antibody or control for 4 weeks (1 week in case of engrafted GBM4) after tumor induction. The resulting tumor-bearing brain tissue was analyzed by FACS for its mouse macrophage surface marker profile. Mice treated with anti-CD47 monoclonal antibodies were found to have an increased number of macrophages with M1 phenotype markers relative to untreated mice (data not shown). The ratio of M1 cell counts (CD80+/total macrophage number) was significantly higher in treated mice (Fig 4A). Further, median fluorescence intensity values of the M1 marker CD80-AF647 within the macrophage gate were significantly elevated in anti-CD47-treated mice (Fig 4B). M2 macrophages, as measured by CD206 fluorescence intensity ratios and cell count normalized to total macrophage count, increased within the tumor as well, but to a much lesser extent (Fig 4B). Further, anti-CD47 treatment was effective in reducing tumor burden of GBM5 xenografted mice as measured by bioluminescence and resulted in a significant survival benefit (Fig 4C).

## Discussion

Recently, we have shown that use of blocking anti-CD47 monoclonal antibodies and peptides are common therapeutic modalities disrupting the innate immune checkpoint by which cells prevent macrophage-mediated phagocytosis [19, 23, 32]. Elimination of tumor cells by anti-CD47 antibodies may occur through a variety of mechanisms which include both phagocytic [19, 33, 34] and non-phagocytic tumor cell killing by neutrophils and NK cells [35, 36]. While the humanized anti-CD47 antibody (Hu5F9-G4) used in this study has been shown to block the CD47-SIRPα interaction and induce phagocytosis of leukemia cells[26] we can not rule out the possibility of Fc mediated opsonization as human macrophages express high affinity Fc receptor FcRγ1. Here, we show that M1 macrophages display a larger phagocytic response towards GBM than M2 macrophages upon tumor cell opsonization and/or CD47-SIRPα disruption *in vitro*. This was observed using both mouse and human macrophages, however, the phagocytic response by both species after anti-CD47 treatment varied by the targeted tumor cell line and the individual macrophage batch used per experiment. CD47-expression was high in all of the analyzed samples and CD47 expression did not positively correlate with phagocytic activity in the tested cell lines. One tumor cell line, GBM4, had a particularly high CD47 expression and displayed a lower baseline and antibody induced phagocytosis. Whether this observation can be extrapolated towards an inverse correlation between phagocytic activity and CD47 expression level remains speculative. Given that CD47 expression levels were not greatly different across most tumor lines, we believe that donor-related Sirp expression-levels may have partially contributed to the range of baseline- and induced-phagocytosis strength toward GBM samples within macrophage subtypes. Alternatively, the balancing effect of the various other “eat me” and don’t eat me” signals in an individual tumor line could contribute to this phenomenon. As another possible explanation, Sirpα-polymorphisms among the different (individual) donor macrophages could have differential levels of phagocytosis as it has been previously shown that polymorphism in SIRPα controls CD47 binding and autoimmunity[37]. It has also been shown that SIRPα polymorphisms controls phagocytosis of apoptotic cells [38, 39] hence we speculate that SIRPα polymorphism could function as a potential biomarker for patient selection for anti-CD47 immunotherapy.

Further, our *in vitro* data showed that mouse M2 and human M2 macrophages were also responsive to CD47-SIRPα disruption, despite this subtype’s tumorigenic growth characteristics and association with worse clinical outcomes. This circumstance might be additionally favorable for GBM patients with high proportions of M2 macrophages within their tumors, as anti-CD47 treatment promotes these macrophages towards tumor phagocytosis. Indeed Willingham et al. showed that macrophages isolated from flank breast, bladder, and liver cancer xenografts phagocytized tumor cells when co-incubated with blocking anti-CD47 antibody *in vitro* [19]. To measure Fc-receptor mediated opsonization effect of Hu5F9 [26] toward GBM, we performed additional phagocytosis assays with nonspecifically Fc-blocked Hu5F9 which consistently sustained elevated levels of GBM phagocytosis. In addition we assessed the effect of pretreatment of either macrophages or tumor cells alone before performing the phagocytosis experiments. The IgG4 isotype antibody did not induce macrophage phagocytosis compared to anti-CD47 treatment. Of note, pretreatment of tumor cells resulted in an equal phagocytosis induction as when adding the antibody directly to the macrophage-tumor cell mix, which is explained by CD47-Sirpα disruption on GBM, whereas pretreatment of macrophages alone was not correlated to higher tumor-cell phagocytosis. While complete Fc-receptor independency of the observed phenomena can not be ruled out, our use of Fc-blocked IgG4 and IgG1 monoclonal antibodies suggests a large proportion of GBM phagocytosis is driven by CD47 inhibition. As seen in here with GBM, prior studies have similarly been unable to use IgG4 isotypes to induce macrophage phagocytosis towards malignancies, reaffirming a Fab-predominant mechanism [26]. Likewise, studies performed by Burger et al. [40] have recently shown that blocking of the CD47-Sirpα interaction by F(ab’)2 fragments was sufficient to induce erythrocyte phagocytosis. In spite of these studies, the varying expression by different tumors of Fc receptor subclasses with their varying binding affinities, along with the potentially synergistic role of Fc-related effects and Hu5F9-G4 antibody therapy, our observed increase of GBM phagocytosis may be a shared contribution of tumor cell opsonization and the disruption of CD47-SIRPα interactions [41, 42]. Hitherto, introduction of additional “eat me” signals in the form of Fc-receptors to promote antibody-dependent cellular cytotoxicity or complement-mediated cytotoxicity in addition to CD47 blockade is currently under investigation as a therapeutic strategy.

Our *in vitro* findings demonstrate for the first time the differential phagocytosis-promoting effects of CD47-SIRPα disruption on mouse and human macrophage polarization subtypes. Various studies have demonstrated the pro-phagocytic effect of anti-CD47 antibodies, albeit in unpolarized macrophages, which we found to be comparable to M2 macrophages [43, 44]. We deciphered the inherent phagocytic potential of macrophage subtypes, isolated from the influence of any variations in microenvironment. However, our findings do not consider the effects of the surrounding cellular and cytokine milieu *in vivo* that may promote tumor viability or modulate other macrophage properties including variations in macrophage subtype polarization states [45–49]. Further, we did not find a polarization shift from M2 to M1 upon anti-CD47 treatment *in vitro*, which might be due to the relatively short exposure of the *in vitro* polarized cells to anti-CD47 treatment and lack of important tumor microenvironment constituent in the *in vitro* situation. Although both M1 and M2 subtypes demonstrate a robust response to CD47-SIRPα disruption, the stronger M1 antitumor response and associated cytokine profile suggest a clinical value from enhancing the M1 macrophage response toward gliomas and other solid tumors [50].

Last, our *in vivo* data show that the macrophage population in the tumor environment is dynamic and shifts toward a pro-inflammatory immune M1 dominant response after anti-CD47 treatment. Importantly, this observation is coupled to a prolonged survival in the treated animals and a significantly reduced tumor burden. In tumors of untreated animals, most TAMs were of the M2 subtype, which is in line with previous studies [51]. Most importantly, we observed that levels of mouse M1 macrophages in the microenvironment of xenografted tumors more than doubled with anti-CD47 treatment, whereas levels of mouse M2 macrophages exhibited a less pronounced increase. Whether or not this increase is caused by an influx of new M1 cells or reflects an augmented macrophage polarization from M2 toward the M1-type remains undetermined. Moreover, the origin of these polarized TAMs remains to be defined, as it might well be that microglial cells of predominantly M2-type represent a major subpopulation within the TAM pool. One might speculate that there is a resulting reprogramming of TAMs, including microglia, toward the M1 phenotype, although it is also possible that anti-CD47 blocking antibodies provided selective environments in the tumor that favor pre-committed M1 macrophages to enter and/or proliferate [51, 52].

Our *in vivo* findings are the first to demonstrate that disruption of the CD47-SIRPα axis and/or Fc mediated tumor cell opsonization results in a more pro-inflammatory immune presence based on the higher M1 content within treated tumors. A potential mechanism for producing a subsequent, local increase in phagocytosis upon anti-CD47 treatment may include cytoskeletal mobilization induced by inhibited SIRPα signaling [53]. Knockdown of SIRPα (and therefore mimicking CD47–SIRPα disruption) has also recently been shown to induce migration of macrophages towards tumor cells, strengthen macrophage survival and propagate a proinflammatory cytokine response via NF-κB signaling, which is consistent with an M1-phenotype [54]. Mechanistically, varying SIRPα signaling levels could therefore be responsible for the observed M1–M2 shift.

Anti-CD47 antibody therapy represents an alternative avenue to promote a pro-inflammatory environment that will enhance the body’s anti-tumor response. Previous efforts have sought to recreate a similar pro-inflammatory microenvironment by personalized vaccinations of irradiated tumor cells transduced with stimulatory B7-2 and GM-CSF genes or, conversely, by inhibiting M2 growth via CSF-1R inhibition [55, 56]. However, anti-CD47 therapy provides a unique means of targeting gliomas by taking advantage of local TAMs and enhancing the M1 macrophage presence and response. The anti-tumor role of macrophages has been a growing field, whereby additional considerations to increase macrophage recruitment and infiltration will likely augment future clinical responses [50, 57, 58]. Finally, we have recently shown that activating macrophages through TLR 3,4,7 leads to a pathway resulting in augmented secretion of truncated phosphorylated calreticulin, which then serves as an “eat me” signal for macrophages guiding these macrophages toward the tumor [59]; switching macrophages to the M1 phenotype should augment this, and therefore anti-CD47 *in vivo* anti-cancer therapies.

We conclude that the promotion of M1 macrophages represents an opportunity to enhance the anti-glioma effect achieved by anti-CD47 monoclonal antibodies, which also promote the classical (M1) phenotype of macrophages.

## Supporting Information

S1 Fig Flow-cytometry gating tree used in polarized phagocytosis assays. (A) Representative flow cytometric analysis demonstrating successful differentiation of CD11b+ F4/80+ mouse macrophages. (B) Representative flow cytometric analysis demonstrating successful differentiation of CD11b+ CD14+ human macrophages. (C) To identify human macrophages, singlets were gated using FSC-W/FSC-H and dead cells were excluded with DAPI. CD11b+ CD14+ cells were considered to be macrophages and further subcharacterized by M1 and M2 specific markers. Tumor cell phagocytosis was assessed using CFSE-labeling. (D) FMO controls for CD14 and CD11b were set to identify the correct macrophage gate for human macrophages. (E) FMO controls for F4/80 and CD11b were set to identify the correct macrophage gate for mouse macrophages
(PDF)

S2 Fig Validation of the phagocytosis assay using calcein stained tumor cells. (A) Live, single CD11b^+^ CD14^+^ human macrophages were gated based on FMO controls. (B) CD11b^+^ CD14^+^ human macrophages were further analyzed. The calcein positive population represents macrophages that have successfully phagocytized tumor cells. Flow cytometry analysis of CD11b^+^ CD14^+^ human macrophages that were not incubated with tumor cells or with Hu5F9-G4 (left panel); analysis of CD11b^+^ CD14^+^ human macrophages incubated with calcein stained tumor cells (middle panel); CD11b^+^ CD14^+^ human macrophages incubated with stained tumor cells pretreated with 10 μg/mL anti Hu5F9-G4 antibody.
(PDF)

S3 Fig Characteristics of mouse M1 and M2 macrophages. (A) Flow-cytometric analysis gated on CD11b+ live singlets on either IFN-γ/LPS or IL-4/IL-13 polarized bone marrow-derived mouse macrophages stained for polarization markers CD80 and CD206. Gates were set based on FMO controls (contour plot overlay). (B) Gene expression analysis by quantitative real-time PCR of mouse M0, M1 and M2 macrophages for N*os1* and *Mrc1*, plotted as relative expression normalized to *Hprt*. Data depicted as mean +/- SD.
(PDF)

S4 Fig Characteristics of human M1 and M2 macrophages (A) Flow cytometric analysis gated on CD11b+ live singlets on either IFN-γ/LPS or IL-4/IL-13 polarized peripheral blood-derived human macrophages stained for polarization markers CD80 and CD163. Gates were set based on FMO controls (contour plot overlay). (B) Luminex-assessed cytokine levels of IL-1ß, TGF-α, IL-10, IL-12P40 and IL-6 in cell culture supernatants of human M0, M1 and M2 macrophages. Values are plotted as an average of median fluorescent intensity compared to M0. Data depicted as mean +/- SD.
(PDF)

S5 Fig Baseline phagocytosis rates of mouse and human M1 and M2 macrophages upon anti-CD47 treatment. (A) Dot plot summarizing the mean rate of phagocytosis for all co-incubated cell lines (GBM1, GBM4 and PGBM1) offered to mouse M1 and M2 macrophages at baseline and after anti-CD47 treatment. (baseline values: M1 10.7% vs. M2 7.3%, p = 0.063, treatment values: M1 26.7% vs. M2 11.7%, respectively, p = 0.066, paired Student’s t-test). (B) Dot plot summarizing the mean rate of phagocytosis for all co-incubated cell lines (GBM1-4 and PGBM1) offered to human M1 and M2 macrophages at baseline and after anti-CD47 treatment. Data depicted as mean +/- SD, each data point represents the mean phagocytosis rate of a single tumor cell line (baseline values: M1 5.2% vs. M2 14.8%, p = 0.067, treated values: M1 52.2% vs. M2 38.8%, p = 0.16, paired Student’s t-test).
(PDF)

S6 Fig Phagocytosis rates of human M0 macrophages, and polarization-dependent phagocytosis of a fetal human neural progenitor cell line by either M1 or M2 human macrophages +/- anti-CD47 treatment. (A) Dot plot summarizing the mean rate of phagocytosis for all co-incubated cell lines (GBM1, GBM4 and PGBM1) offered to human M0 macrophages after anti-CD47 treatment. Data depicted as mean +/- SD, each data point represents the mean phagocytosis rate of a single tumor cell line (p = 0.0006, paired t-test). (B) Differential phagocytosis activity of human M1 or M2 macrophages towards human fetal neural progenitor cells in comparison to GBM1, -/+ anti-CD47 treatment demonstrating that anti-CD47 treatment is specific for tumor cells as rates of phagocytosis were not higher in neural progenitor cells vs. glioblastoma. (C) Cumulative phagocytosis activity of human macrophage subtypes towards human fetal neural progenitor cells, GBM1-4, and PGBM1. Each bar represents a summation of the different mean phagocytosis rates of a single cell line by human M1 and M2 macrophages -/+ anti-CD47 treatment, as denoted by different colors.
(PDF)

S7 Fig CD47 expression of the tested cell lines. Cell lines were incubated with fluorescently labeled anti-CD47 antibodies or isotype control antibodies. Median fluorescent intensities were measured and isotype-corrected (cf. also Table 2).
(PDF)
