# Supplementary material for: Anti-CD47 Treatment Stimulates Phagocytosis of Glioblastoma by M1 and M2 Polarized Macrophages and Promotes M1 Polarized Macrophages In Vivo
Source: PLoS One. 2016 Apr 19;11(4):e0153550. doi: 10.1371/journal.pone.0153550 (PMC4836698; doi:10.1371/journal.pone.0153550)
Supplement: S1 Fig — (A) Representative flow cytometric analysis demonstrating successful differentiation of CD11b+ F4/80+ mouse macrophages. (B) Representative flow cytometric analysis demonstrating successful differentiation of CD11b+ CD14+ human macrophages. (C) To identify human macrophages, singlets were gated using FSC-W/FSC-H and dead cells were excluded with DAPI. CD11b+ CD14+ cells were considered to be macrophages and further subcharacterized by M1 and M2 specific markers. Tumor cell phagocytosis was assessed using CFSE-labeling. (D) FMO controls for CD14 and CD11b were set to identify the correct macrophage gate for human macrophages. (E) FMO controls for F4/80 and CD11b were set to identify the correct macrophage gate for mouse macrophages (PDF) [file pone.0153550.s001.pdf]

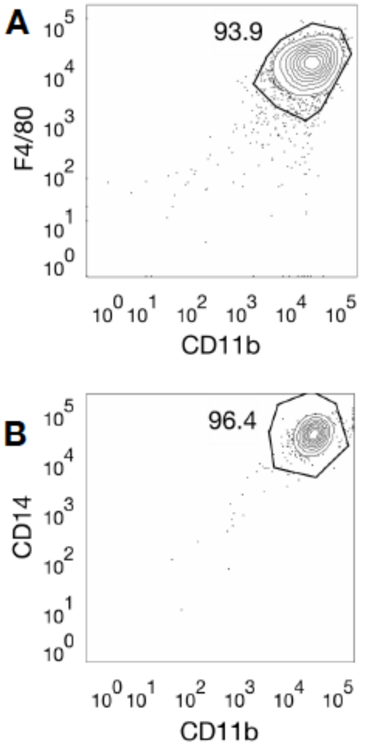

C

Population	Count	Freq. of Parent	Freq. of Total
Ungated	50609	100	100
Debris Exclusion	44161	87.3	87.3
SSC Singlets	41485	93.9	82.0
FSC Singlets	40727	98.2	80.5
Live-Dead	37933	93.1	75.0
CD11b-CD14	4793	12.6	9.47
Phagocytosis	579	12.1	1.14

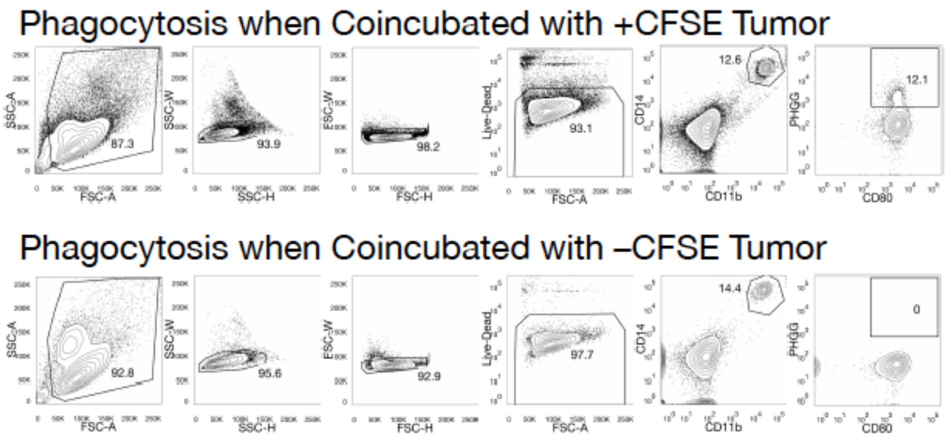

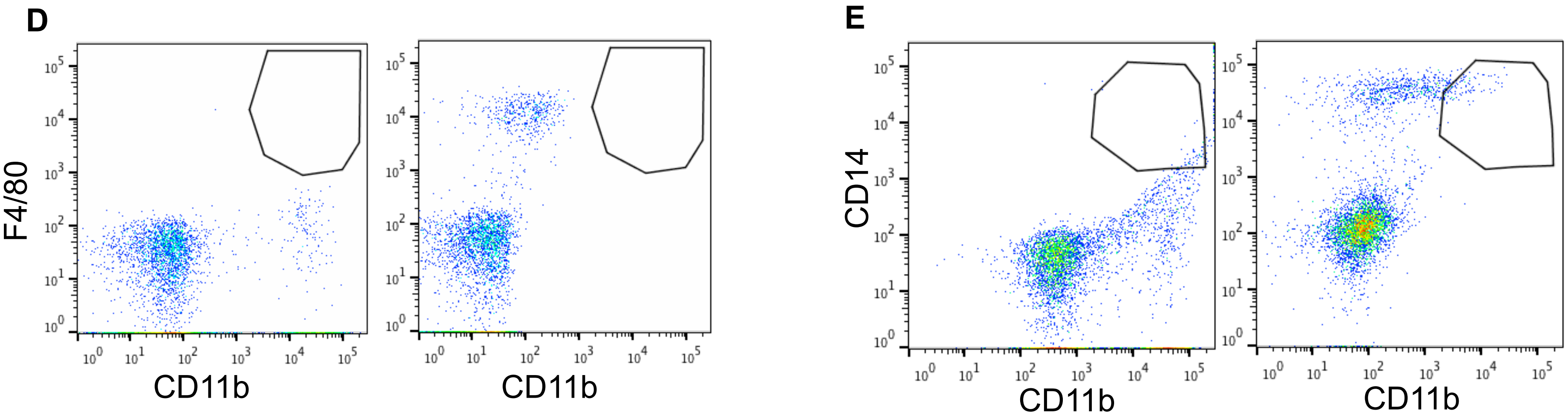

S1 Figure
