# Supplementary figures and images for: Anti-CD47 Treatment Stimulates Phagocytosis of Glioblastoma by M1 and M2 Polarized Macrophages and Promotes M1 Polarized Macrophages In Vivo
Source: PLoS One. 2016 Apr 19;11(4):e0153550. doi: 10.1371/journal.pone.0153550 (PMC4836698; doi:10.1371/journal.pone.0153550)

**A**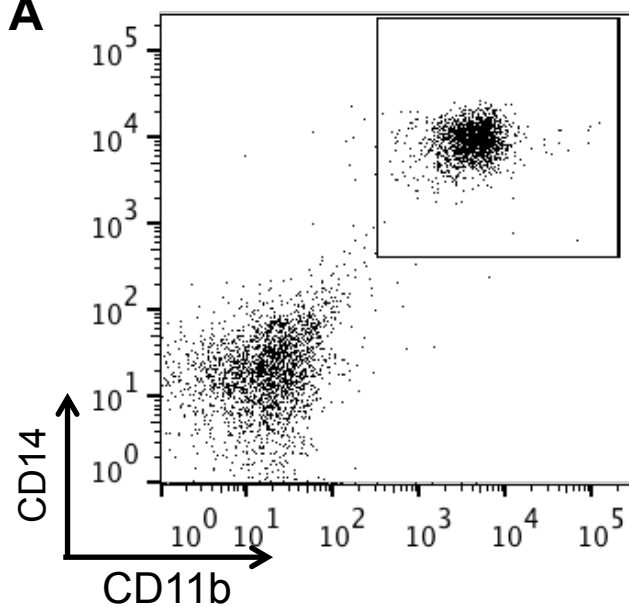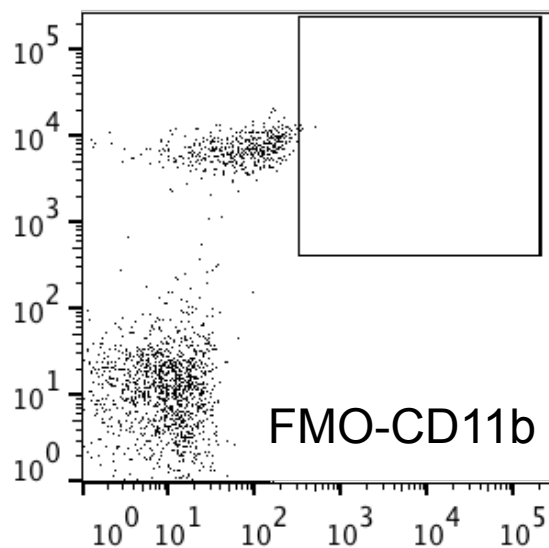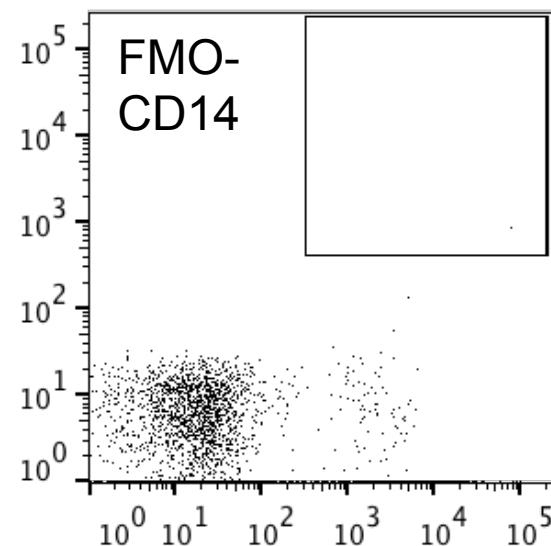**B**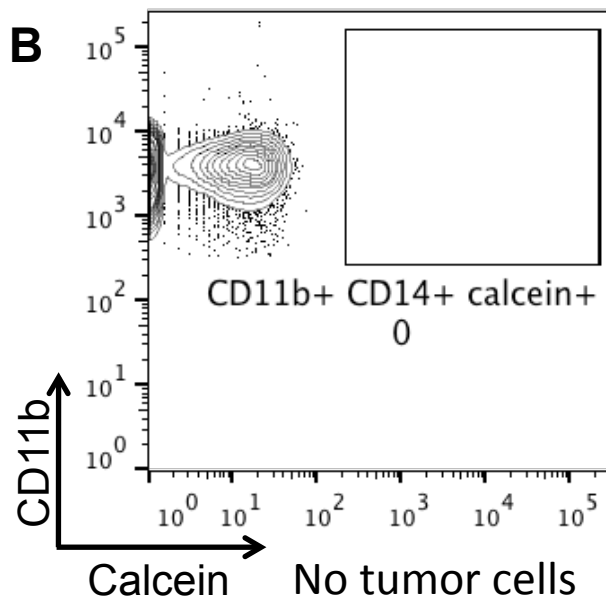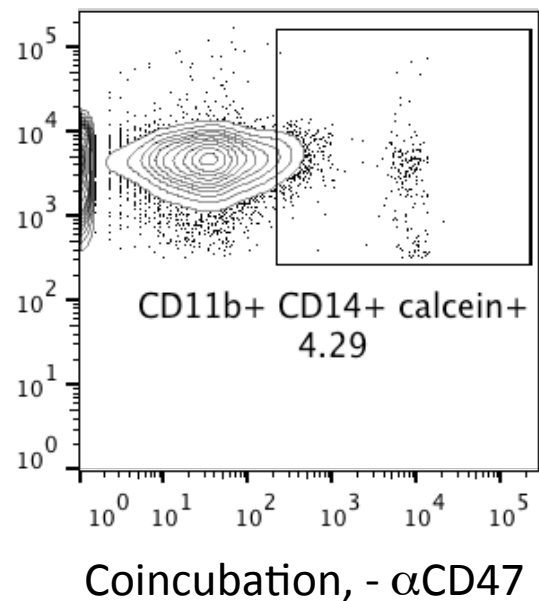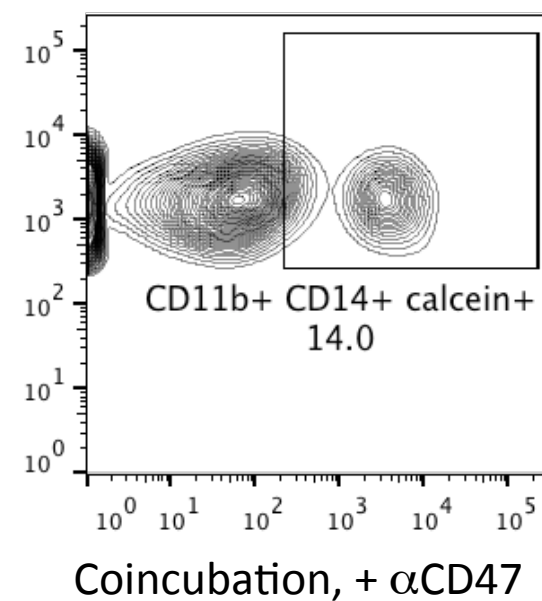**S2 Figure**

Supplement: S2 Fig — (A) Live, single CD11b+ CD14+ human macrophages were gated based on FMO controls. (B) CD11b+ CD14+ human macrophages were further analyzed. The calcein positive population represents macrophages that have successfully phagocytized tumor cells. Flow cytometry analysis of CD11b+ CD14+ human macrophages that were not incubated with tumor cells or with Hu5F9-G4 (left panel); analysis of CD11b+ CD14+ human macrophages incubated with calcein stained tumor cells (middle panel); CD11b+ CD14+ human macrophages incubated with stained tumor cells pretreated with 10 μg/mL anti Hu5F9-G4 antibody. (PDF) [file pone.0153550.s002.pdf]

**A**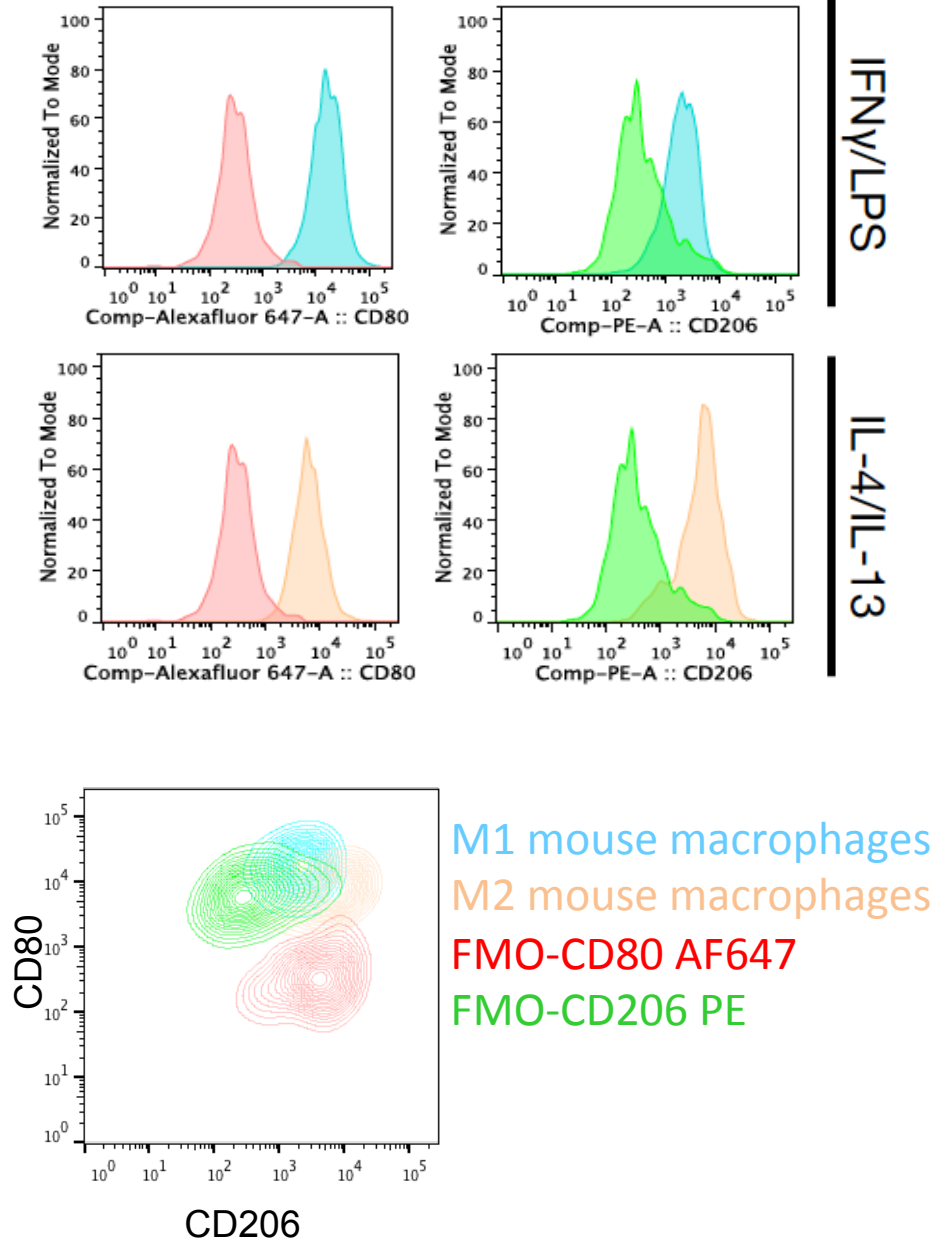**B**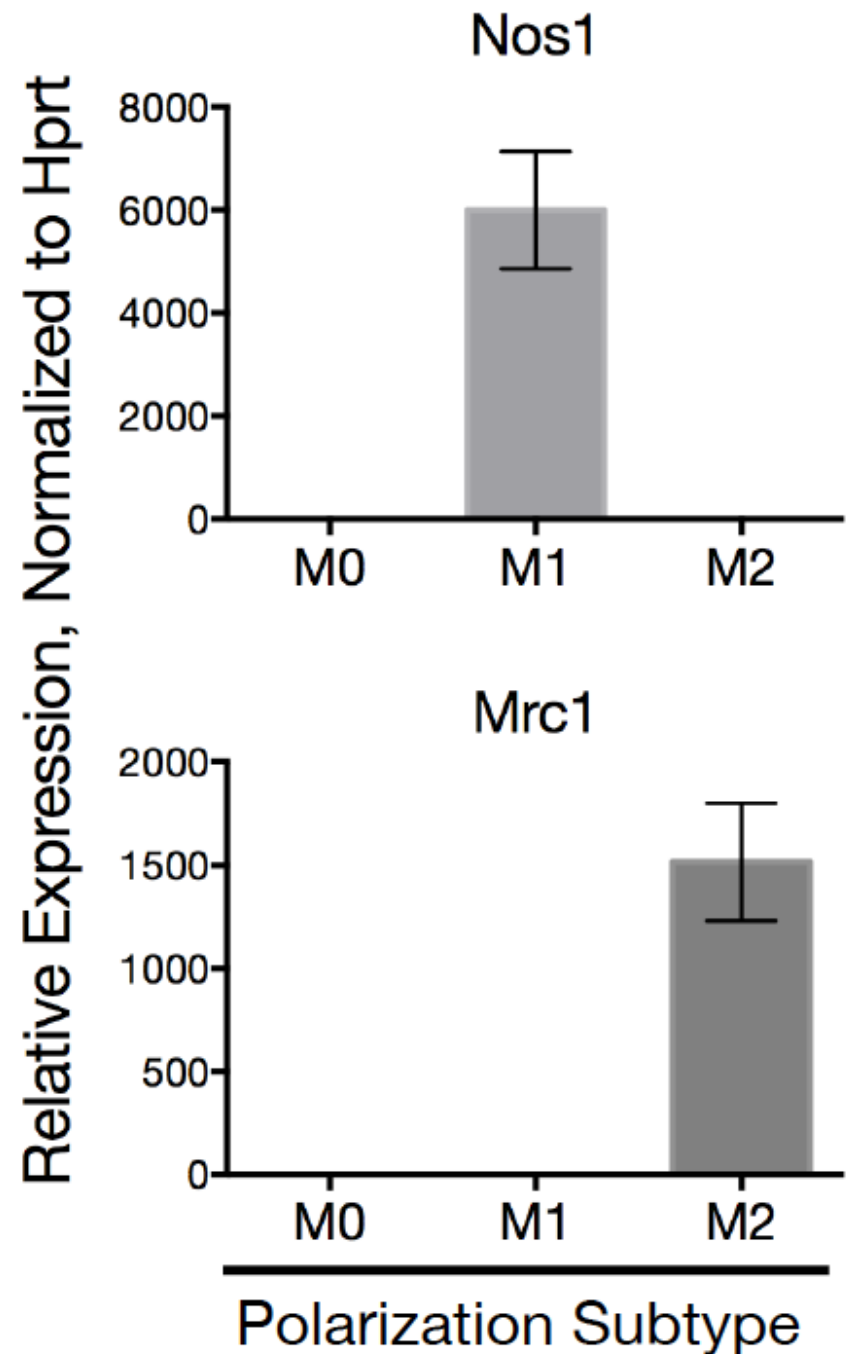**S3 Figure**

Supplement: S3 Fig — (A) Flow-cytometric analysis gated on CD11b+ live singlets on either IFN-γ/LPS or IL-4/IL-13 polarized bone marrow-derived mouse macrophages stained for polarization markers CD80 and CD206. Gates were set based on FMO controls (contour plot overlay). (B) Gene expression analysis by quantitative real-time PCR of mouse M0, M1 and M2 macrophages for Nos1 and Mrc1, plotted as relative expression normalized to Hprt. Data depicted as mean +/- SD. (PDF) [file pone.0153550.s003.pdf]

**A**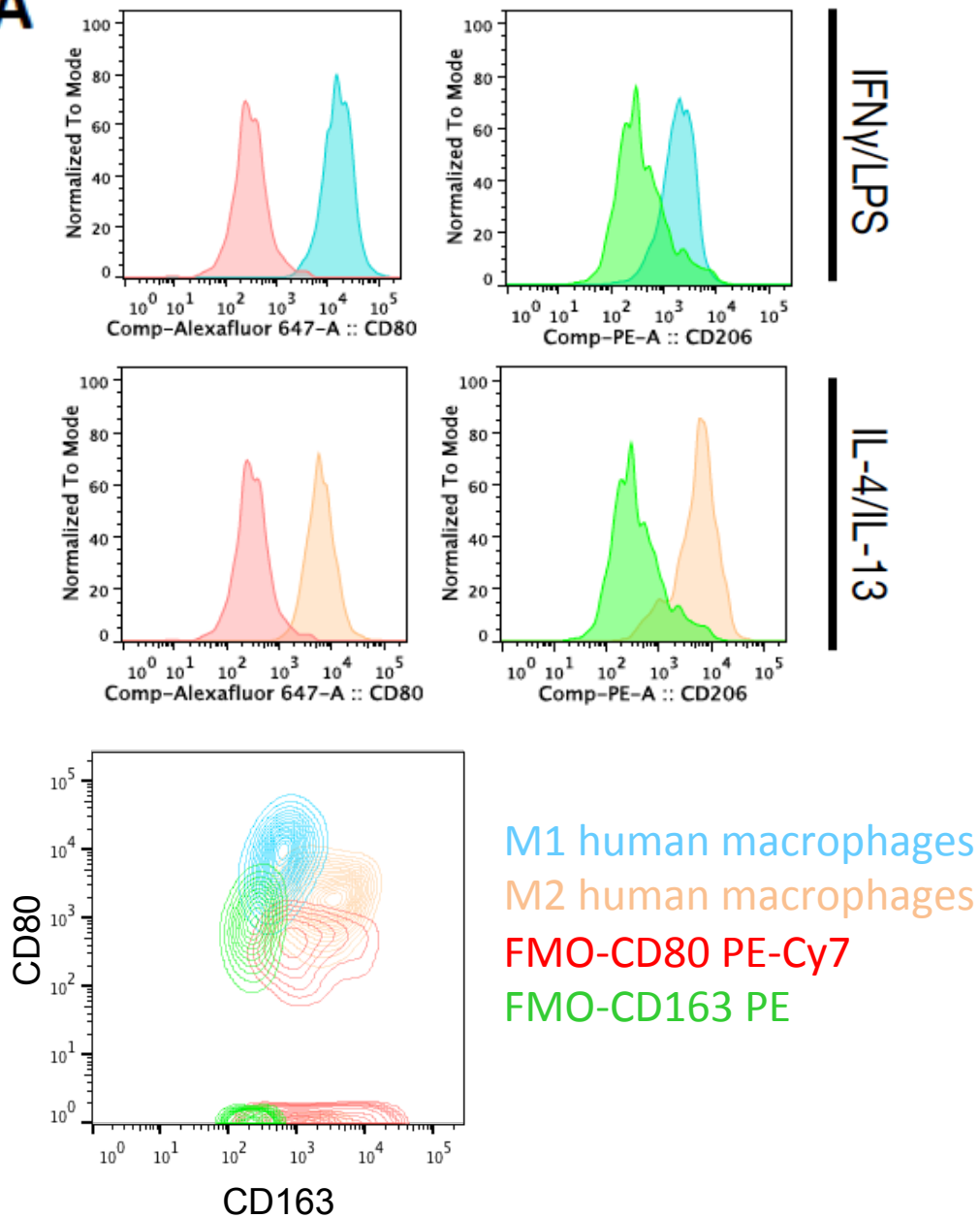**B**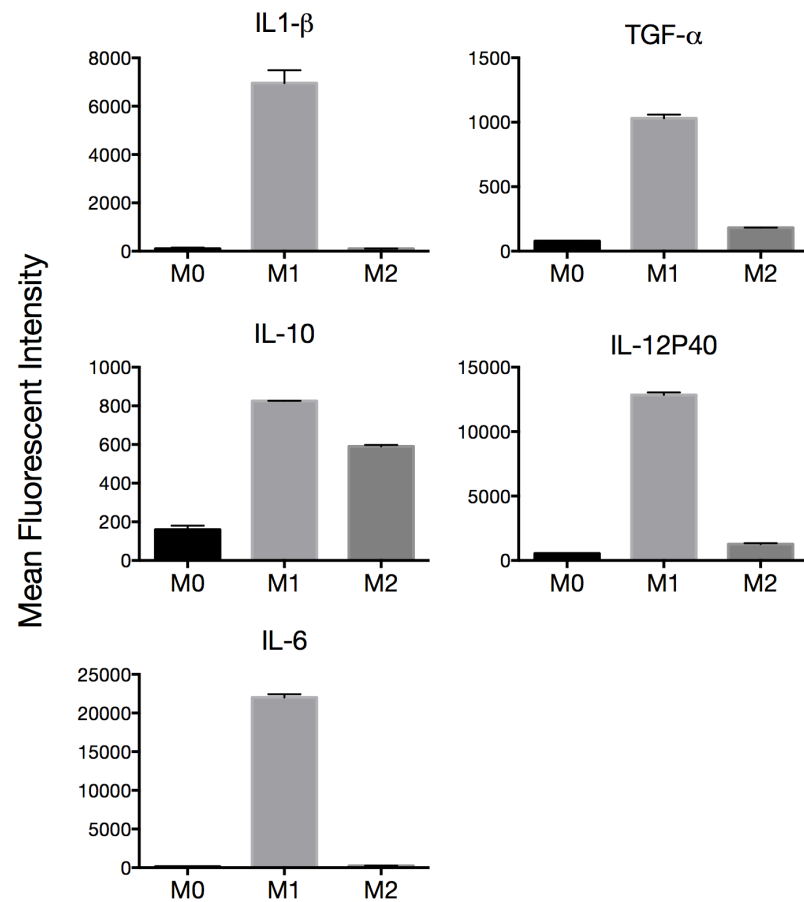**S4 Figure**

Supplement: S4 Fig — (A) Flow cytometric analysis gated on CD11b+ live singlets on either IFN-γ/LPS or IL-4/IL-13 polarized peripheral blood-derived human macrophages stained for polarization markers CD80 and CD163. Gates were set based on FMO controls (contour plot overlay). (B) Luminex-assessed cytokine levels of IL-1ß, TGF-α, IL-10, IL-12P40 and IL-6 in cell culture supernatants of human M0, M1 and M2 macrophages. Values are plotted as an average of median fluorescent intensity compared to M0. Data depicted as mean +/- SD. (PDF) [file pone.0153550.s004.pdf]

**A**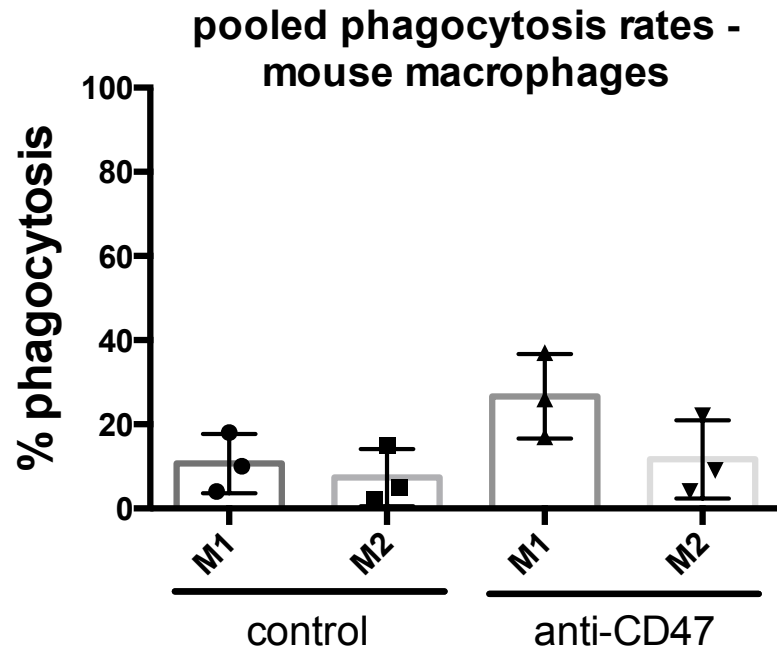**B**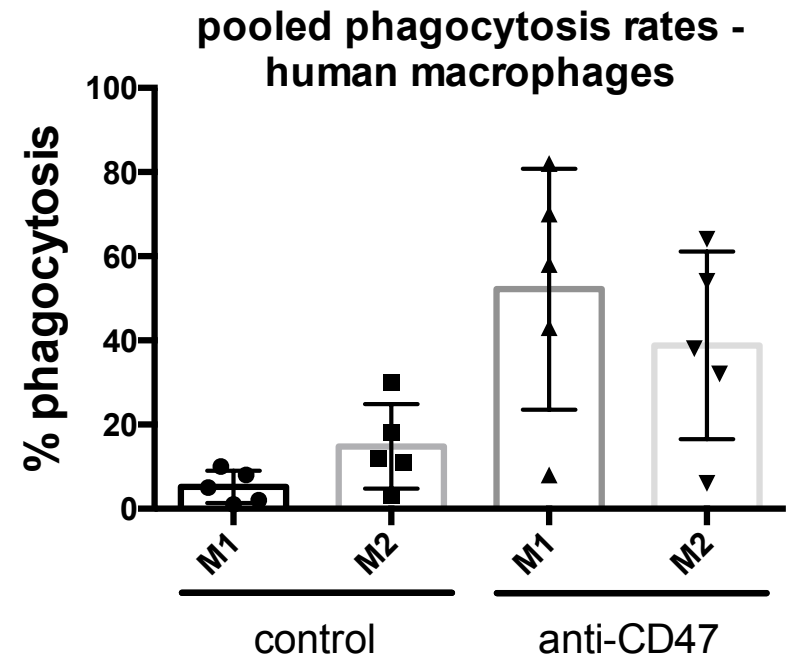

Supplement: S5 Fig — (A) Dot plot summarizing the mean rate of phagocytosis for all co-incubated cell lines (GBM1, GBM4 and PGBM1) offered to mouse M1 and M2 macrophages at baseline and after anti-CD47 treatment. (baseline values: M1 10.7% vs. M2 7.3%, p = 0.063, treatment values: M1 26.7% vs. M2 11.7%, respectively, p = 0.066, paired Student’s t-test). (B) Dot plot summarizing the mean rate of phagocytosis for all co-incubated cell lines (GBM1-4 and PGBM1) offered to human M1 and M2 macrophages at baseline and after anti-CD47 treatment. Data depicted as mean +/- SD, each data point represents the mean phagocytosis rate of a single tumor cell line (baseline values: M1 5.2% vs. M2 14.8%, p = 0.067, treated values: M1 52.2% vs. M2 38.8%, p = 0.16, paired Student’s t-test). (PDF) [file pone.0153550.s005.pdf]

**A**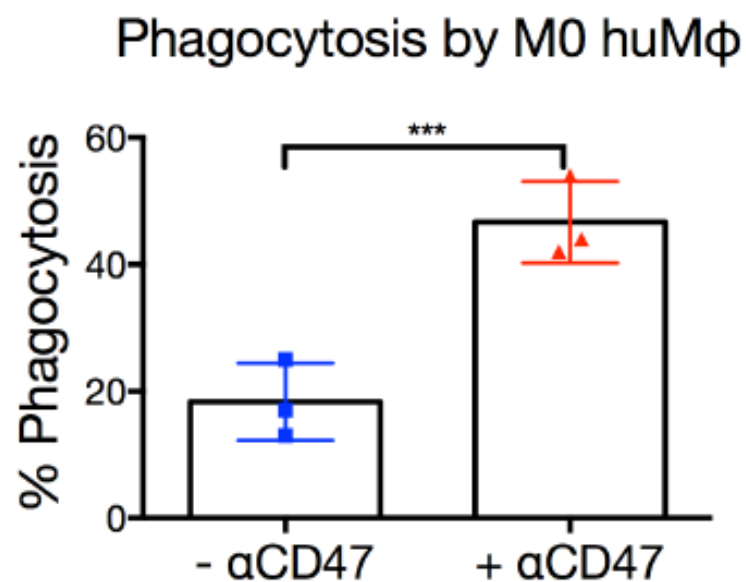**B**

Comparative Macrophage Activity Towards Neural Progenitor Cells and GBM1

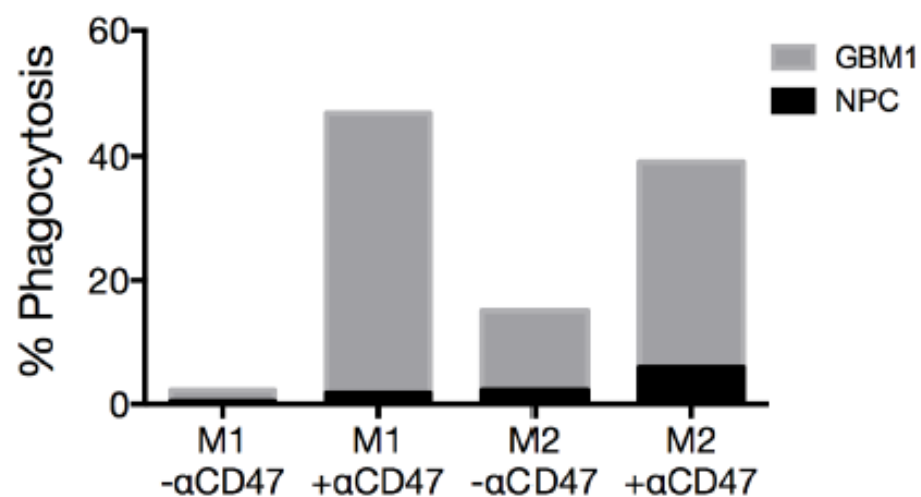**C**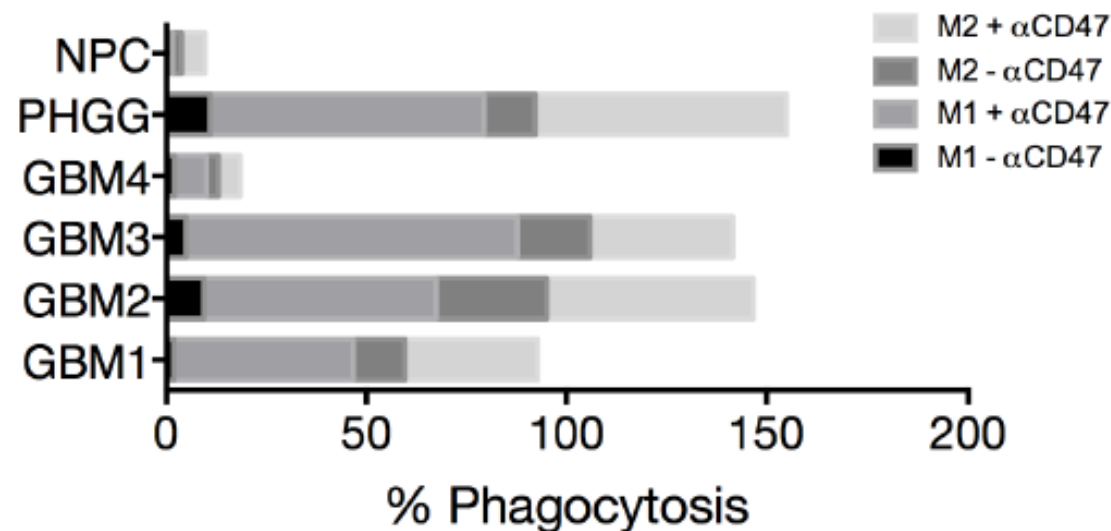

**S6 Figure**

Supplement: S6 Fig — (A) Dot plot summarizing the mean rate of phagocytosis for all co-incubated cell lines (GBM1, GBM4 and PGBM1) offered to human M0 macrophages after anti-CD47 treatment. Data depicted as mean +/- SD, each data point represents the mean phagocytosis rate of a single tumor cell line (p = 0.0006, paired t-test). (B) Differential phagocytosis activity of human M1 or M2 macrophages towards human fetal neural progenitor cells in comparison to GBM1, -/+ anti-CD47 treatment demonstrating that anti-CD47 treatment is specific for tumor cells as rates of phagocytosis were not higher in neural progenitor cells vs. glioblastoma. (C) Cumulative phagocytosis activity of human macrophage subtypes towards human fetal neural progenitor cells, GBM1-4, and PGBM1. Each bar represents a summation of the different mean phagocytosis rates of a single cell line by human M1 and M2 macrophages -/+ anti-CD47 treatment, as denoted by different colors. (PDF) [file pone.0153550.s006.pdf]

# CD47-Expression of tumor cell lines

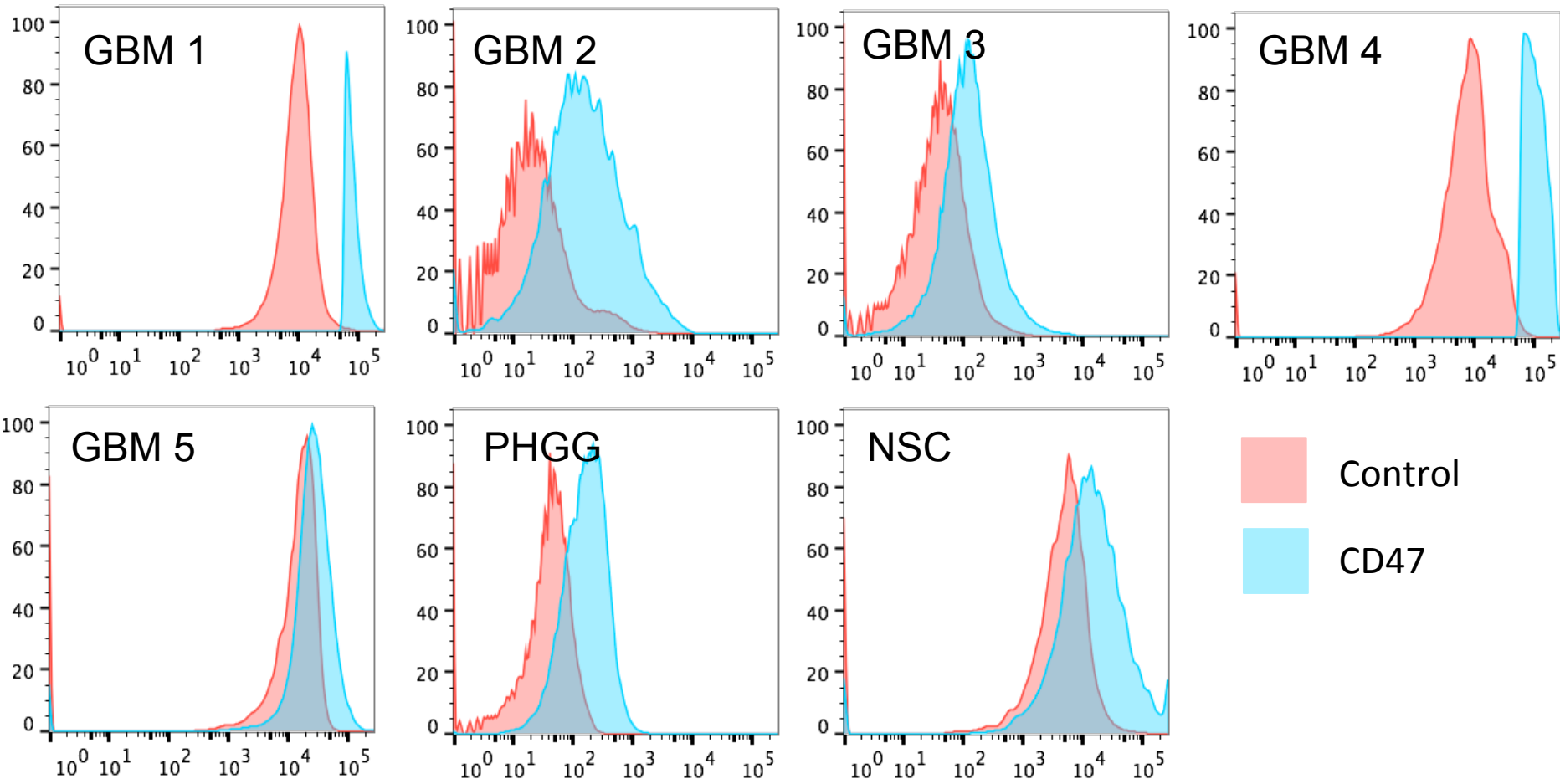

S7 Figure

Supplement: S7 Fig — Cell lines were incubated with fluorescently labeled anti-CD47 antibodies or isotype control antibodies. Median fluorescent intensities were measured and isotype-corrected (cf. also Table 2). (PDF) [file pone.0153550.s007.pdf]
